# Posttranslational Regulation of Botulinum Neurotoxin Production in Clostridium botulinum Hall A-*hyper*

**DOI:** 10.1128/mSphere.00328-21

**Published:** 2021-08-04

**Authors:** Heather N’te Inzalaco, William H. Tepp, Chase Fredrick, Marite Bradshaw, Eric A. Johnson, Sabine Pellett

**Affiliations:** a Department of Bacteriology, University of Wisconsin-Madison, Madison, Wisconsin, USA; University of Maryland Medical Center

**Keywords:** *Clostridium botulinum*, Hall A-*hyper*, botulinum neurotoxin, BoNT/A, botulinum toxin complex, arginine, pH, metalloprotease, posttranslational

## Abstract

Botulinum neurotoxins (BoNTs) are the most toxic substances known to humankind and are the causative agents of the neuroparalytic disease botulism. Despite the overall importance of BoNTs in public health and safety, as a bioterrorism concern, and in pharmaceutical development, little is known about the molecular mechanisms mediating BoNT stability and degradation in various environments. Previous studies using Clostridium botulinum strain ATCC 3502 revealed that high levels of arginine (20 g/liter) repressed BoNT production approximately 1,000-fold. In the present study, the mechanisms of toxin reduction in arginine-enriched cultures of C. botulinum strain Hall A-*hyper*, which we have previously genetically manipulated using ClosTron technology, were explored. Cultures were grown in toxin production medium (TPM) and TPM enriched with arginine. Cultures were analyzed for growth (optical density at 600 nm [OD_600_]), changes in pH, and BoNT formation and stability. Our data indicate that arginine enrichment of C. botulinum strain Hall A-*hyper* cultures results in a pH shift that induces pH-dependent posttranslational control mechanisms. We further show that independent of arginine, maintenance of an acidic culture pH during growth of C. botulinum strain Hall A-*hyper* plays a central role in toxin stability and that an extracellular metalloprotease produced by the culture results in BoNT degradation at pH levels between ⁓6.5 and 8.0.

**IMPORTANCE** Botulinum neurotoxin (BoNT) is a public health and bioterrorism concern as well as an important and widely used pharmaceutical, yet the regulation of its synthesis by BoNT-producing clostridia is not well understood. This paper highlights the role of environmentally controlled posttranslational regulatory mechanisms influencing processing and stability of biologically active BoNTs produced by C. botulinum. The results of this work will help enhance public health and safety measures and our ability to evaluate safety risks of novel BoNTs and improve production and quality of BoNTs for pharmaceutical use.

## INTRODUCTION

The obligate anaerobe bacterium Clostridium botulinum is a genetically diverse species of Gram-positive, spore-forming bacilli grouped together by their ability to produce potent botulinum neurotoxins (BoNTs) (1–4). BoNTs are potent protein toxins that cause the neuroparalytic disease botulism in humans and vertebrate animals (5–7). BoNTs are AB-type endopeptidases with a catalytically active 50-kDa light chain (LC) domain and a 100-kDa heavy chain (HC) domain linked by a disulfide bond. The HC facilitates specific neuronal cell binding and entry, while the LC is a zinc-dependent endopeptidase that specifically targets and cleaves SNARE (*N*-ethylmaleimide sensitive factor [NSF] attachment protein receptor) proteins found at motor neuron junctions (8, 9). This cleavage results in blocking neurotransmission of primarily cholinergic nerves, causing the characteristic flaccid paralysis that can last from several days to months, depending on BoNT serotype, subtype, and dose (5, 10–14).

BoNTs are categorized into seven immunologically and functionally distinct serotypes (A to G) and over 40 known subtypes (1, 4, 10, 13). Collectively, C. botulinum bacteria encompass four distinct genetic and physiological groups, termed groups I to IV. Group I consists of proteolytic strains that produce BoNT/A, BoNT/B, and BoNT/F and are predominantly responsible for botulism outbreaks in humans, which can result from ingestion of contaminated foods, infection of wounds or infant intestines, or intentional exposure (1, 3, 4, 15). Group II strains, which are also responsible for botulism outbreaks in humans, are categorized as nonproteolytic and produce BoNT/B, BoNT/E, or BoNT/F, while group III strains affect nonhuman animals and consist of strains that form BoNT/C and BoNT/D (1, 16). BoNT/G-producing strains are categorized in group IV and have not been associated with either human or animal botulism (1, 17).

BoNT/A1 causes the most severe and long-lasting botulism, whereas BoNT/B1 and other serotypes generally have lower potency and a shorter duration. Due to its unique properties, BoNT/A1 is widely employed in the medical field as a unique, long-lasting, local muscle paralytic and for an increasing number of other neurologic disorders (18–22).

All BoNTs are produced as complexes with one or several nontoxic proteins, including nontoxic nonhemagglutinin (NTNH), several hemagglutinin (HA) proteins, or proteins of unknown function (ORFX and p47) (23). The nontoxigenic complex proteins appear to protect the BoNTs from environmental insults and have been proposed to aid in uptake of the toxins from the intestinal environment (24–27). The genes encoding the BoNTs and nontoxic complex proteins are located within a single gene cluster, in which NTNH is encoded in one operon together with BoNT and can be transcribed as mono- or polycistronic (28), and the HA or ORFX proteins are encoded in a second operon that is transcribed in the opposite direction. In addition to the toxin complex genes, most, but not all, BoNT gene clusters also encode an alternative sigma factor protein termed BotR (29, 30). BoNTs are expressed as a 150-kDa single-chain protein, which in proteolytic (group I) C. botulinum is then proteolytically processed by cleavage between the LC and HC, leading to the disulfide-bonded active dichain protein (31).

In spite of the significance of BoNTs as potent toxins for humans and animals, they are also widely used biopharmaceuticals. Little is known about molecular and metabolic processes regulating production of these toxins. This is in part due to the challenges of studying toxin production in such a diverse species and under difficult anaerobic culture conditions and in part due to the regulations and restrictions associated with investigating these potent toxins and organisms producing them, which are classified in the United States as Tier 1 Category A Select Agents. Of the studies conducted so far on regulation of BoNT formation, most have focused on elucidating transcriptional control mechanisms, which are commonly employed by pathogenic bacteria to regulate production of virulence factors. Such studies indicate that several factors regulate BoNT gene transcription to a minor extent (mostly 2- to 5-fold), including the global transcription regulator CodY and the sigma factor BotR present in some, but not all, BoNT gene clusters, and by two-component systems and quorum sensing (14, 29, 30, 32–34). It is also widely recognized that environmental stimuli and nutritional factors are central for growth, sporulation, lysis, and metabolism in toxigenic clostridia (6). For example, in group I C. botulinum, a nitrogen, a carbohydrate source, and some amino acids, including arginine, are critical for optimal growth and BoNT production (35–38).

Despite arginine being an essential amino acid, previous studies showed that an excess of arginine in the medium dramatically decreased BoNT formation up to 1,000-fold (35, 39), which is much higher than many other transcriptional control mechanisms that have been demonstrated in toxin-forming pathogens. Similarly, some Clostridioides difficile strains have poor growth but enhanced toxin production in medium with low arginine levels (40).

In this study, the mechanism underlying the strong suppression of toxin formation by arginine in proteolytic C. botulinum strain Hall A-*hyper* was investigated. Arginine is an important nutrient for group I C. botulinum strains, which catabolize arginine via an arginine deiminase (ADI) pathway resulting in the formation of metabolites including citrulline, ornithine, carbamoyl phosphate, ATP, and ammonia (37, 38, 41). Arginine deiminase pathways, consisting of a cluster of genes termed *arc* genes, have also been demonstrated in numerous other organisms, including clostridial species (37, 41–44). In this study, homologous genes comprising an arginine catabolism pathway were identified in the genome of C. botulinum strain Hall A-*hyper*. Results from mutational analyses of several of these genes and from nutritional analyses indicated that the reduction of toxicity was not a direct effect of arginine or one of its metabolites but rather appeared to be related to a metabolically driven increase in culture pH during growth in excess arginine. Further studies showed pH-dependent proteolytic degradation of BoNT in culture media, indicating a strong pH-dependent, posttranslational control mechanism for toxin production by C. botulinum strain Hall A-*hyper*, which in turn is controlled by metabolic changes during growth.

## RESULTS

### Clostridium botulinum strain Hall A-*hyper* contains all genes necessary for a complete arginine deaminase pathway.

**FIG 1:**
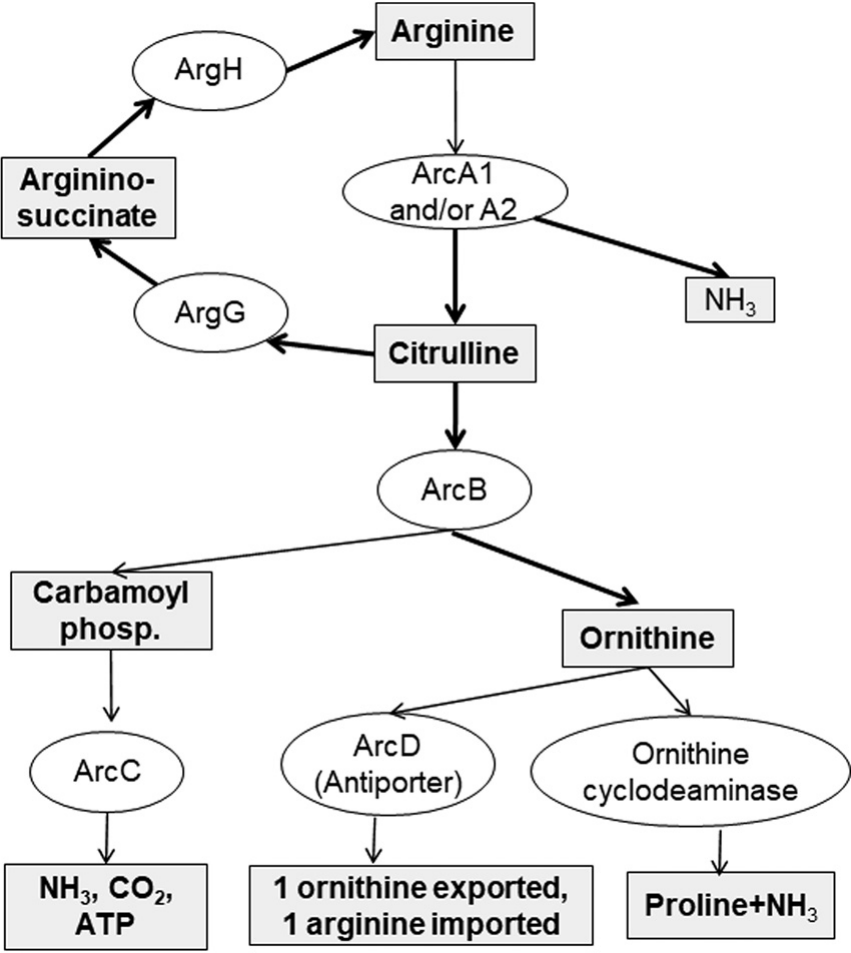
Putative arginine deiminase pathway of C. botulinum strain Hall A-*hyper*. Diagram of the main metabolites and enzymes involved in the arginine deiminase pathway found in the sequenced genome of C. botulinum Hall A-*hyper*.

The genome sequence of C. botulinum strain Hall A-*hyper* (45) was examined for the presence of homologs to genes that are known to be involved in arginine catabolism pathways in other organisms including several clostridia (37, 41–44). The annotated genome of C. botulinum strain Hall A-*hyper* includes several *arc* genes central to the arginine deaminase pathway (37, 41–43), as well as an ornithine catabolism gene (ornithine cyclodeaminase, CLC2447), and two genes (*argG*, CLC2544, and *argH*, CLC2543) controlling arginine formation from citrulline. Catabolism of arginine through the ADI pathway results in the production of ammonia, citrulline, ornithine, carbamoyl phosphate, CO_2_, and ATP, in addition to other metabolites produced from these products (35, 37, 41–43). Based on these data together with the identified putative involved genes in strain Hall A-*hyper*, an arginine catabolism pathway for this strain was constructed (Fig. 1).

### Botulinum neurotoxin production by C. botulinum strain Hall A-*hyper* is strongly suppressed by catabolism of arginine or citrulline during growth.

**FIG 2:**
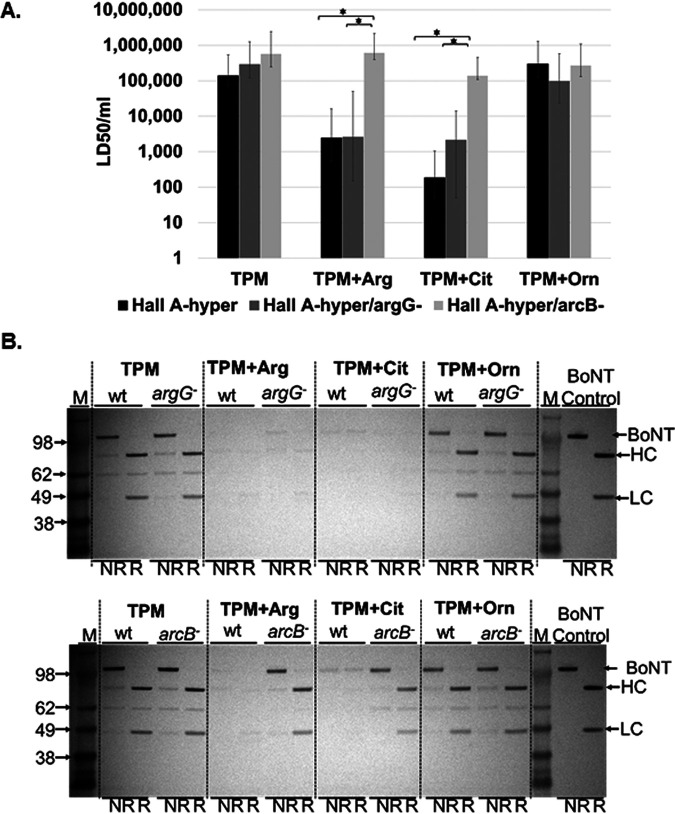
Repression of BoNT production in C. botulinum and derivatives by arginine and metabolites. C. botulinum strains Hall A-*hyper* (wt), Hall A-*hyper*/ArcB-, and Hall A-*hyper*/ArgG- were grown in toxin production medium (TPM) supplemented with arginine, citrulline, or ornithine. (A) Toxicity of trypsinized culture supernatants was determined by the mouse bioassay and is shown as mouse LD_50_/ml. Values represent the mean from four mice and standard deviation. Asterisks represent a statistically significant difference (*P *< 0.05). (B) Western blot assays analyzing whole-culture samples (including cell pellets and supernatant) using anti-BoNT/A specific antibody. Control is 50 ng of purified BoNT/A1. Marker is SeeBlue Plus2 prestained protein standard (Life Technologies), and the molecular weight of the marker bands is indicated on the left in kDa. NR indicates a nonreduced sample, and R indicates a sample reduced with 100 mM dithiothreitol (DTT). Numbers at left are molecular masses in kilodaltons.

Previously, we had observed an ∼1,000-fold suppression of active BoNT production in a C. botulinum type A strain, ATCC 3502, by 2% (0.115 M) arginine, a concentration present in arginine-rich foods such as some seeds, nuts, and meats, added to complex medium (toxin production medium [TPM]) (39). Similar suppression was observed in C. botulinum strain Hall A-*hyper* (Fig. 2). In order to investigate the role of arginine catabolism in toxin suppression in C. botulinum strain Hall A-*hyper*, the genes encoding two key enzymes in the arginine catabolism pathway, ArcB (catalyzing catabolism of citrulline to ornithine and carbamoyl phosphate) and ArgG (catalyzing conversion of citrulline to arginine through a feedback loop), were inactivated using ClosTron technology, yielding strains Hall A-*hyper*-*argG*- and -*arcB*- (46, 47). Attempts to inactivate *arcC* (catalyzing conversion of carbamoyl phosphate to NH_3_, CO_2_, and ATP) using two different ClosTron constructs resulted in lack of survival of colonies, indicating that *arcC* is an essential gene in C. botulinum Hall A-*hyper*. C. botulinum strain Hall A-*hyper* and the two derivatives *argG*- and *arcB*- were examined in complex TPM and TPM supplemented with 2% arginine. Mouse bioassay of culture supernatants and Western blot analysis of whole-culture samples at day 6 of incubation showed that neither mutation affected BoNT production or growth characteristics in TPM (Fig. 2A and B). However, supplementation of the medium with arginine or citrulline resulted in strong suppression of BoNT production in the wild type (wt) and the *argG*- strain but not in the *arcB*- strain (Fig. 2B). In addition, a strong increase in cell density for the wt and *argG*- strains was observed in the presence of 2% arginine and a limited increase was observed in the presence of 2% citrulline, as determined by an ∼2.5- and 1.5-fold increase in optical density at 600 nm (OD_600_), respectively (see Fig. S1 in the supplemental material). Supplementation of the medium with an equal concentration of the (downstream) catabolite ornithine, on the other hand, did not affect BoNT production in either the wt or the mutant strains and did not increase cell density (Fig. 2B and Fig. S1).

10.1128/mSphere.00328-21.1 FIG S1 Growth of C. botulinum Hall A-*hyper* and Hall A-*hyper*/*argG*- and Hall A-*hyper*/*arcB*-. C. botulinum Hall A-*hyper* wt and *argG*- or *arcB*- strains were grown in TPM, TPM with 2% arginine, TPM with 2% citrulline, or TPM with 2% ornithine. Growth was analyzed by OD_600_ readings. Data are average values from three biological replicates, and error bars indicate the standard deviation for each time point. Download FIG S1, TIF file, 1.2 MB. Copyright © 2021 Inzalaco et al. 2021 Inzalaco et al. https://creativecommons.org/licenses/by/4.0/ This content is distributed under the terms of the Creative Commons Attribution 4.0 International license.

Taken together, these data indicate that arginine and citrulline must be catabolized at least past the step involving the *arcB* gene to result in suppression of BoNT production and that the presence of 2% arginine or citrulline in a strain lacking a functional *arcB* gene, which is required to further metabolize citrulline, does not result in BoNT suppression. This indicates that neither arginine nor citrulline is likely to play a direct role in BoNT suppression, while their catabolism causes the suppression. Since ornithine, a catabolite of arginine or citrulline, had no effect on toxin production, the data further suggest that ornithine or its catabolites are also not directly responsible for BoNT suppression by arginine and citrulline. Thus, other catabolites in the ADI pathway, such as carbamoyl phosphate, ATP, or ammonia may be involved in BoNT regulation (Fig. 1). Since attempts to disrupt the *arcC* gene were unsuccessful, as it likely is an essential gene, this hypothesis could not be tested directly.

**FIG 3:**
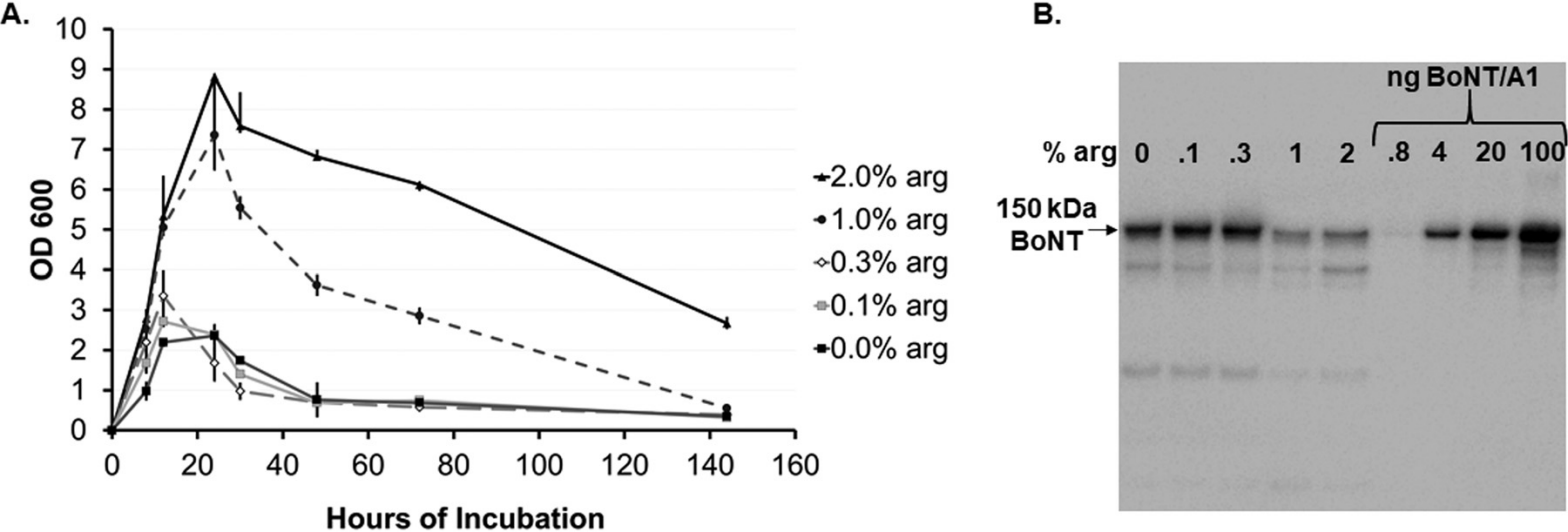
Concentration-dependent suppression of BoNT production in C. botulinum strain Hall A-*hyper* by arginine. (A) Growth (OD_600_) of C. botulinum strain Hall A-*hyper* grown in TPM or TPM with the indicated arginine concentrations for up to 6 days. Data are average and standard deviation from three biological replicates at each time point. (B) Representative Western blot image (one of triplicate samples) of culture supernatants at 6 days. Culture supernatants were analyzed by Western blotting using polyclonal anti-BoNT/A1 antibody. Four known amounts of BoNT/A1 were used as a toxin standard indicated as nanograms loaded. For both panels, arginine has been abbreviated as arg.

In order to determine the minimal concentration of arginine required for BoNT repression in C. botulinum strain Hall A-*hyper*, triplicate cultures were grown in TPM supplemented with either 0%, 0.1%, 0.3%, 1.0%, or 2% arginine. Analysis of day 6 culture samples by Western blotting showed a significant decrease in BoNT production in medium enriched with 1% and 2% arginine compared to cultures grown in medium with arginine concentrations below ≤0.3% (*P* < 0.003) (Fig. 3B). Similarly, absorbance at OD_600_ showed a divergence for peak growth and lysis patterns (Fig. 3A) as previously observed for other cultures that resulted in BoNT suppression (Fig. S1). Cultures enriched with arginine at 1% and 2% grew to a greater density with OD_600_ values of 7.4 and 8.8, respectively, and did not reach full lysis until after day 6. Cultures enriched with ≤0.3% arginine grew as the nonenriched culture did to densities of about 2.5 to 3 OD_600_ and lysed rapidly within 24 h post-stationary phase (Fig. 3A). These data indicate that the suppression of BoNT production and enhanced cell density by arginine requires at least 1% arginine with no apparent gradual dose response.

### BoNT suppression by arginine in C. botulinum Hall A-*hyper* occurs posttranscriptionally.

**FIG 4:**
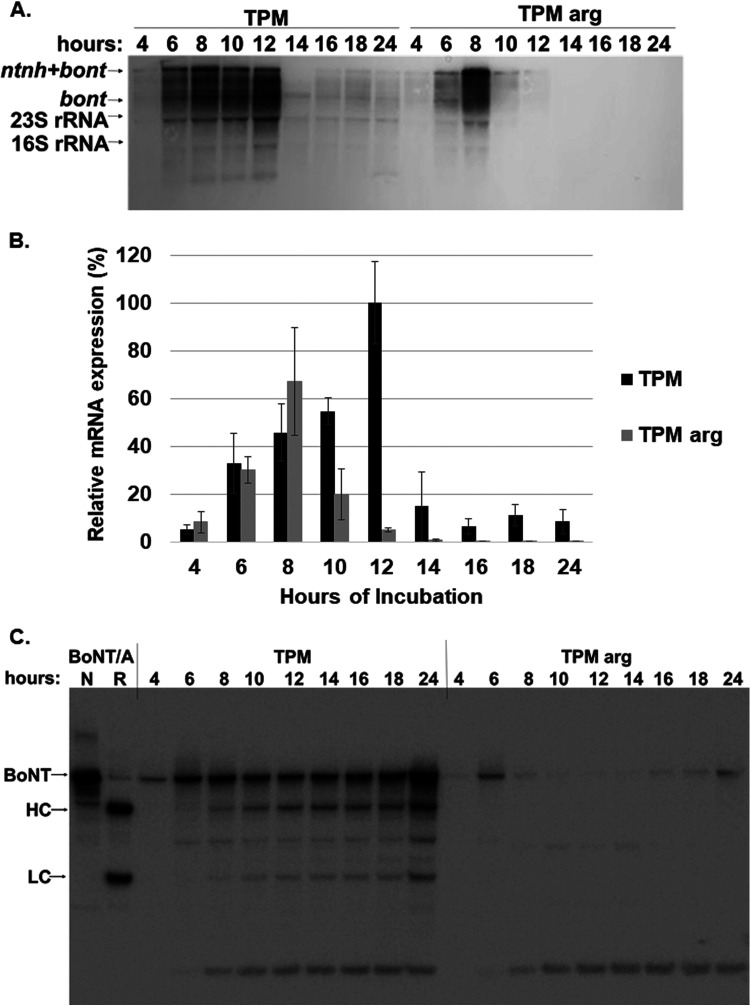
Protein and mRNA levels of *bont/a* produced by C. botulinum Hall A-*hyper* in TPM with 2% arginine. C. botulinum strain Hall A-*hyper* cultures (*n* = 3) were grown in TPM or TPM with 2% arginine for up to 24 h, and protein levels and mRNA levels of BoNT/A were examined by Western blotting and Northern blotting, respectively. (A) Representative Northern blot assay of total RNA collected at the indicated times. The Northern membrane was hybridized to specific *bont/a1* antisense RNA probe. The apparent ∼7.5-kb *ntnh+bont* bicistronic and ∼3.9-kb *bont* monocistronic mRNA bands are shown. The sizes of the 2.9-kb 23S and 1.5-kb 16S rRNA are indicated and appear as white bands on the blot. (B) Quantitative depiction of relative mRNA levels of *ntnh*+*bont* and *bont* at the indicated time points normalized to the averaged levels at 12 h for cultures grown in TPM from triplicate Northern blot assays. Data are average and standard deviation from triplicate biologic replicates. (C) Representative Western blot image of nonreduced whole-culture samples of the same cultures used for the Northern blot assay, analyzed by Western blotting using polyclonal anti-BoNT/A1 antibody. The sample collection times are indicated.

Since BoNT production by C. botulinum involves a series of intricate and only partially understood steps of transcription, processing, complex assembly, and proteolytic activation, we next sought to determine whether arginine catabolism affects BoNT production at the transcriptional level. Northern blot analyses of total RNA of C. botulinum strain Hall A-*hyper* grown in TPM or TPM with 2% arginine for up to 24 h revealed distinct ∼7.5-kb (corresponding to bicistronic *ntnh+bont* mRNA) and ∼3.9-kb (corresponding to monocistronic *bont*) bands with a specific *bont/a* mRNA probe (Fig. 4A). Peak mRNA levels were observed at about 8 to 12 h, consistent with previously reported results showing detectable *bont* mRNA during late log and early stationary phase only (34). The patterns for relative expression of the NTNH-BoNT-linked genes were similar between the two culture treatments, with quantitative data (including bicistronic and monocistronic mRNA) of triplicate plots showing no significant difference in overall mRNA levels but a slightly earlier peak in medium containing arginine, which corresponds to an earlier peak in cell density (OD_600_ values not shown) (Fig. 4B).

Western blot analysis of cell lysates of the same cultures, on the other hand, showed significantly stronger BoNT formation for the culture grown in TPM than for the culture grown in TPM with arginine, where BoNT bands were barely detectable (Fig. 4C). In addition, while BoNT levels in the culture grown in TPM appear to increase over 24 h, the BoNT levels in the culture grown in TPM with arginine decreased after log phase. These results indicate that arginine does not directly influence transcriptional control of BoNT production.

### Suppression of BoNT production by C. botulinum Hall A-*hyper* in medium containing 2% arginine is correlated with increased culture pH.

**FIG 5:**
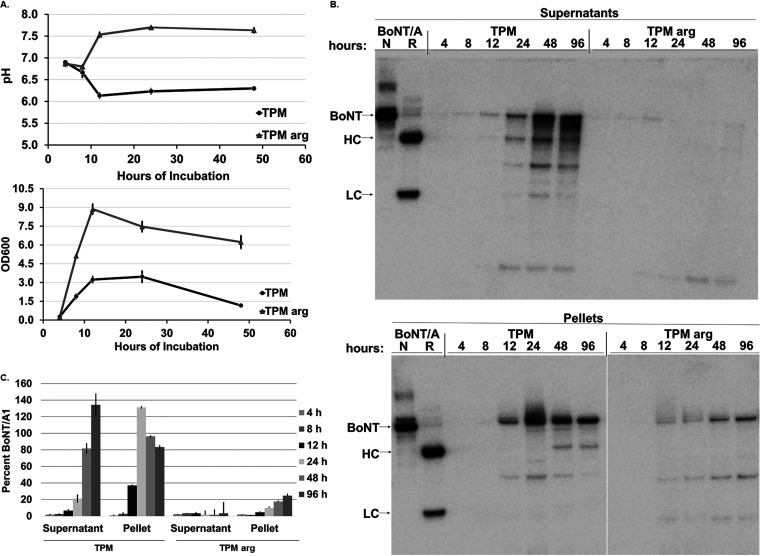
Culture pH, growth, and BoNT/A production by C. botulinum strain Hall A-*hyper* grown for 4 days in TPM or TPM with 2% arginine. (A) Culture pH and growth (OD_600_) of C. botulinum strain Hall A-*hyper* grown in TPM or TPM with 2% arginine for up to 4 days. The pH values are shown for up to 2 days, after which time they did not change significantly anymore. Average and standard deviation from three biologic replicates are shown. (B) Representative Western blot images of C. botulinum strain Hall A-*hyper* culture grown in TPM or TPM with 2% arginine for up to 4 days. Culture supernatants and cell pellets were collected at the indicated time points, and the same volumes were analyzed by Western blotting using polyclonal anti-BoNT/A antibody. The full-length BoNT, heavy chain (HC), and light chain (LC) are indicated. (C) Quantitative comparison of BoNT/A detected by Western blotting (*n* = 3). Approximate percent BoNT was quantified for all arginine-treated samples based on the calibrated average of three 100-ng BoNT/A1 toxin standards loaded on the same gel. Data are mean values from three biological replicates, and error bars indicate ±SD from the mean.

BoNT produced by C. botulinum is predominantly released into the culture supernatant during the lysis stage of cell growth. Since cell lysis is delayed in C. botulinum strain Hall A-*hyper* grown in TPM with 2% arginine, BoNT levels in culture supernatant and cell pellets of cultures grown in TPM or TPM with 2% arginine for up to 4 days were examined by Western blotting. In cultures grown in TPM, increasing amounts of BoNT in culture supernatants were observed over the 4-day time period, and amounts in cell pellets increased earlier up to a peak at 24 h, followed by a slow decrease (Fig. 5B and C). The increase of toxin in culture supernatant with correlating decrease in cell pellets after 24 h is in agreement with BoNT release from the cells during the lysis phase, although a surprisingly large amount of toxin remained associated with the cell pellet even after 96 h. In the cultures grown in TPM with 2% arginine, there was very little detectable toxin in the culture supernatant as expected. In addition, significantly less toxin was associated with the cell pellet compared to that of TPM cultures (*P* < 0.001), with slowly increasing amounts throughout the 4-day study period (Fig. 5B and C). This indicates that the decrease of BoNT in culture supernatant is not due to lack of release of the toxin from the cells due to slower lysis but that BoNT formation in cells is also decreased.

To further investigate the mechanism underlying the suppression of BoNT production by arginine, culture pH was examined during growth of these cultures. While the pH of the culture grown in TPM steadily decreased during log phase from the starting pH of about 7 to an acidic pH of about 6, there consistently was a shift in pH to alkaline values (∼7.5 to 7.8) at 8 h with 2% arginine supplementation (Fig. 5A). The same divergence of pH values was also observed for cultures grown in medium enriched with 1% and 2% arginine compared to cultures enriched with arginine at or below 0.3% and in the cultures used to prepare RNA samples for the Northern blot analyses (Fig. S2). Upon additional review, a correlation of BoNT suppression and increased culture pH was also observed for cultures of C. botulinum strain Hall A-*hyper* wt, Hall A-*hyper argG*-, and Hall A-*hyper arcB*- in TPM and TPM supplemented with arginine, citrulline, or ornithine (Table S1). Together, these data strongly suggest a correlation of increased culture pH with alkaline levels and BoNT suppression.

10.1128/mSphere.00328-21.2 FIG S2 Culture pH of C. botulinum Hall A-*hyper* cultures during growth. (A) Culture pH during growth at various arginine concentrations (Fig. 5). (B) Culture pH during growth in TPM or TPM with 2% arginine of the cultures used for the mRNA and toxin expression study (Fig. 4). Download FIG S2, TIF file, 0.2 MB. Copyright © 2021 Inzalaco et al. 2021 Inzalaco et al. https://creativecommons.org/licenses/by/4.0/ This content is distributed under the terms of the Creative Commons Attribution 4.0 International license.

10.1128/mSphere.00328-21.3 TABLE S1 List of pH values of C. botulinum strains Hall A-*hyper*, Hall A-*hyper argG*-, and Hall A-*hyper arcB*- grown in TPM or TPM supplemented with 2% arginine (Arg), citrulline (Cit), or ornithine (Orn). The culture pH values at 24 h and 144 h of culture at 37°C in an anaerobic environment are shown. Download Table S1, DOCX file, 0.01 MB. Copyright © 2021 Inzalaco et al. 2021 Inzalaco et al. https://creativecommons.org/licenses/by/4.0/ This content is distributed under the terms of the Creative Commons Attribution 4.0 International license.

### Suppression of BoNT production by C. botulinum Hall A-*hyper* in medium containing 2% arginine is due to a shift to alkaline culture pH.

**FIG 6:**
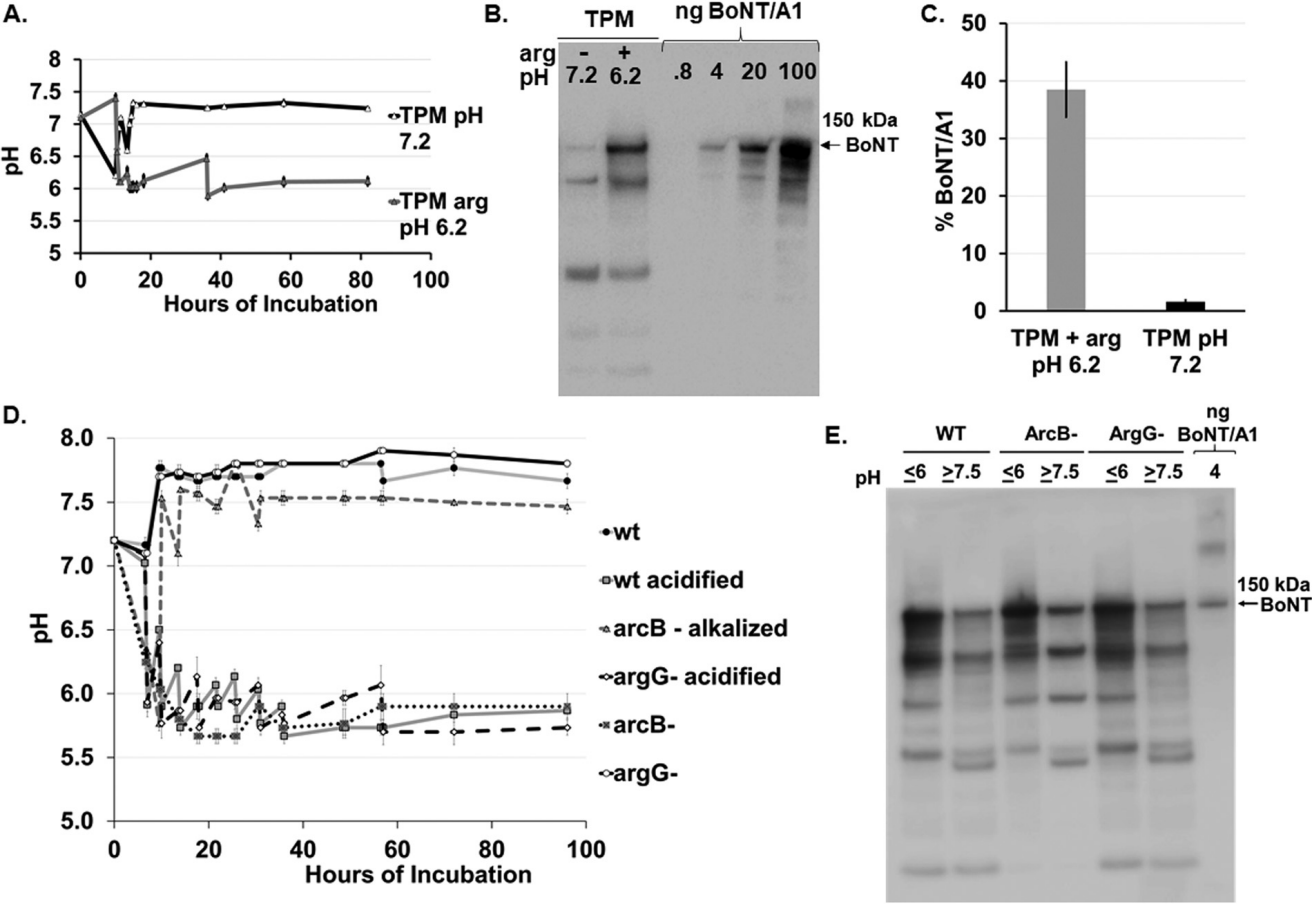
BoNT/A production by C. botulinum Hall A-*hyper* wt, *arcB*-, or *argG*- grown in TPM or TPM with 2% arginine with pH levels reversed manually. (A) Culture pH of C. botulinum strain Hall A-*hyper* grown in TPM or TPM with 2% arginine for up to 82 h. Culture pH was adjusted manually by adding either 5 M HCl or 4 M NaOH at 10 and 18 h of incubation to maintain desired (reversed) pH values for each medium type. Samples growing in TPM were kept at a pH of 7.3 to 7.4, whereas samples growing in TPM plus 2% arginine were kept at pH 6.0 to 6.2. Data are average and standard deviations from three biological replicates. (B) Representative Western blot image of nonreduced whole-culture samples collected at 82 h. Culture samples were lysed in SDS sample buffer, heated to 70°C for 10 min, and analyzed by Western blotting using polyclonal anti-BoNT/A antibody. Purified BoNT/A1 was used as a toxin standard, with the nanograms loaded indicated. (C) Quantitative comparison of Western blot data from panel B (*n* = 3). The amount of BoNT in nanograms is determined by the standard curve from the 4 concentrations of purified BoNT/A1 used as a standard as seen in panel B. Data are mean values from three biological replicates, and error bars indicate ±SD from the mean. (D) Cultures of Hall A-*hyper* (wt) (only two sets), Hall A-*hyper arcB*-, or Hall A-*hyper argG*- were grown in TPM with 2% arginine for 96 h and either had pH manually reversed (those labeled as acidified or alkalized) or did not have pH adjusted (*n* = 3). (E) Representative Western blot of BoNT/A1 produced by pH-adjusted or nonadjusted cultures of Hall A-*hyper* (wt) (only two sets), Hall A-*hyper arcB*-, or Hall A-*hyper argG*- grown in TPM with 2% arginine for 96 h. Culture samples were lysed in SDS sample buffer, heated to 70°C for 10 min, and analyzed by Western blotting using polyclonal anti-BoNT/A antibody. Purified BoNT/A1 was used as a toxin size standard.

Since arginine catabolism produces an excess of free ammonia (Fig. 1), we hypothesized that the increase in ammonia leads to an increase in culture pH, which in turn might lead to BoNT instability and degradation. To test this hypothesis, BoNT production was monitored in cultures of C. botulinum grown in TPM or TPM with 2% arginine for 82 h while manually reversing the pH pattern normally observed for cultures grown in TPM or TPM with 2% arginine. Since addition of various buffers to the culture medium was not sufficient to prevent a change in pH during growth, medium was adjusted manually by repeated addition of HCl or NaOH after the log phase of growth, at which time the shift in pH naturally occurs in these cultures (Fig. 5A and Fig. S2). C. botulinum strain Hall A-*hyper* wt cultures grown in TPM were adjusted to maintain an average pH of ⁓7.4, while cultures grown in TPM with arginine were adjusted to an average pH of ⁓6.2 (Fig. 6A). Western blot analysis of 82-h nonreduced culture samples clearly indicated that suppression of BoNT/A was correlated with pH and not with the presence of arginine in medium (Fig. 6B and C).

This hypothesis was further examined by repeating the same assay reversing the pH in parallel with cultures of C. botulinum strains Hall A-*hyper*, Hall A-*hyper arcB*-, and Hall A-*hyper argG*- grown in TPM with 2% arginine. Cultures of strain Hall A-*hyper arcB*- dropped to an acidic pH of ∼5.7 to 6.0 at around 8 h of growth as expected, and a second set of the same cultures was manually adjusted to an alkaline pH of ∼7.5 to 7.8. Cultures of Hall A-*hyper* wt or Hall A-*hyper argG*-, on the other hand, increased in pH at around 8 h to an alkaline pH of about 7.8, and a second set of cultures for each strain was manually adjusted to an acidic pH of 5.7 to 6.0. Western blot analysis of cultures at 96 h indicated that the previously observed BoNT/A suppression in strain Hall A-*hyper arcB*- and wt cultures supplemented with 2% arginine (Fig. 2B) was reversed by adjusting culture pH to an alkaline value (∼7.5) (Fig. 6D and E). Conversely, BoNT/A stability was induced for strain Hall A-*hyper argG*- in TPM cultures with 2% arginine by maintaining an acidic pH below 6.0. These data confirm that BoNT stability in TPM with 2% arginine is dependent on culture pH and is independent of the presence of arginine, suggesting an important role of metabolically induced culture pH in BoNT stability.

### BoNT is proteolytically degraded in culture supernatants by a metalloprotease in a pH-dependent manner.

**FIG 7:**
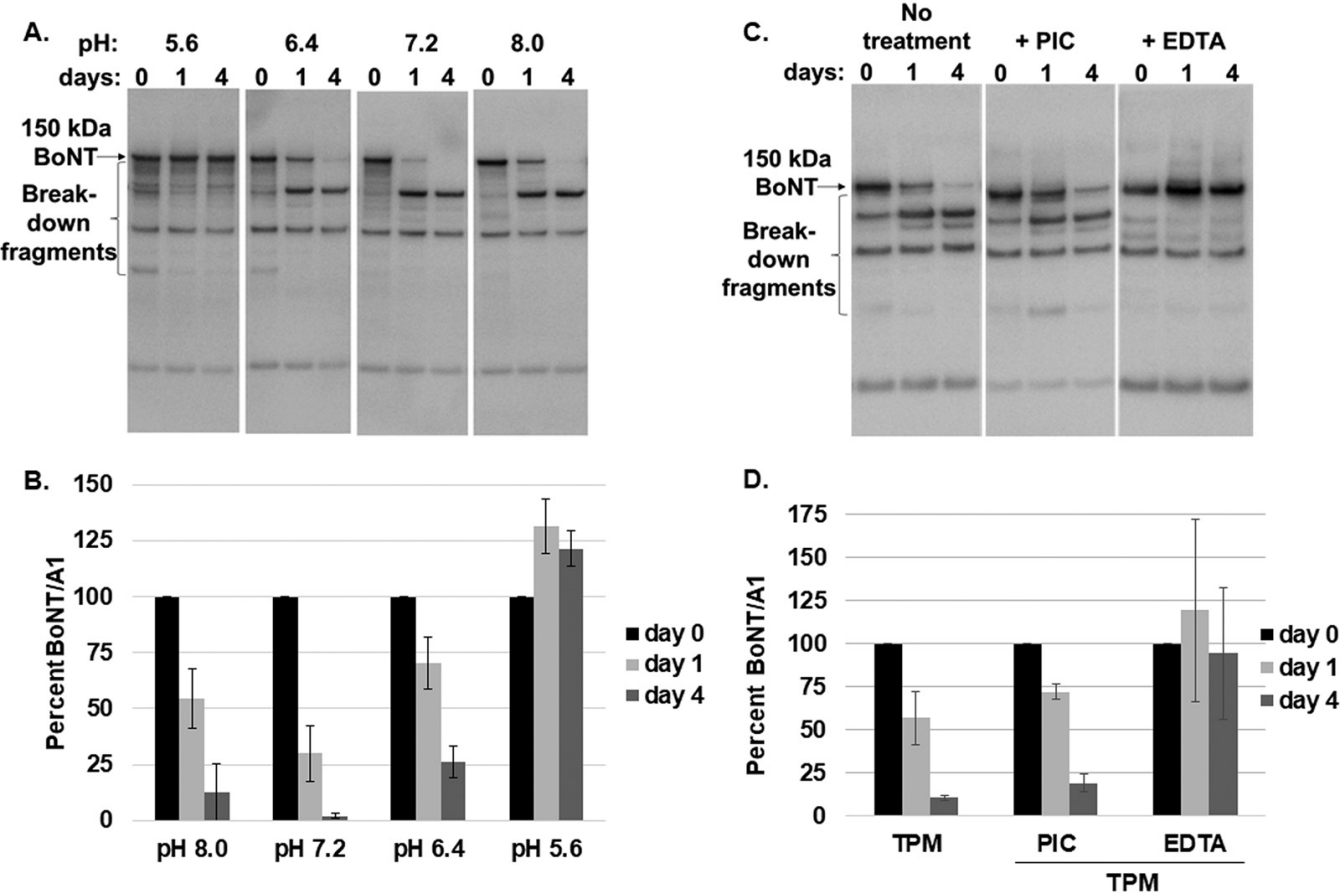
Proteolytic degradation of BoNT/A1 in culture medium of C. botulinum Hall A*-hyper*. Spent culture supernatants of 48-h C. botulinum Hall A-*hyper* cultures (*n* = 3) were recovered and sterile filtered. (A and B) The spent culture supernatant was adjusted to pH 5.6, 6.4, 7.2, or 8.0 and incubated for up to 4 days at 37°C. (A) Representative Western blot of samples at days 0, 1, and 4 probed with polyclonal anti-BoNT/A antibody. Equal volumes of nonreduced culture supernatants were loaded. (B) Quantitative depiction of densitometry data from triplicate Western blots of samples at various pH levels. Average and standard deviations from triplicate samples are shown. For each pH group of samples, the 0-day time point was defined as 100% BoNT/A (starting amount). (C and D) Spent culture supernatant was adjusted to pH 7.2; either EDTA-free protease inhibitor cocktail (PIC), EDTA, or nothing was added; and samples were incubated for up to 4 days at 37°C (*n* = 3). (C) Representative Western blot of samples at days 0, 1, and 4 probed with polyclonal anti-BoNT/A antibody. Equal volumes of nonreduced culture supernatants were loaded. (D) Quantitative depiction of densitometry data from triplicate Western blots. For each pH group of samples, the 0-day time point was defined as 100% BoNT/A (starting amount), and only full-length BoNT/A was quantified.

To further explore the mechanism by which pH influences the stability of BoNT, *in vitro* experiments were conducted that examined BoNT stability in spent culture supernatants from C. botulinum strain Hall A-*hyper* grown in TPM for 48 h. The culture supernatants were pH adjusted to a range of values, sterile filtered, and incubated at 37°C for up to 4 days, the time period for which the C. botulinum production culture is usually grown before toxin harvest. BoNT complex added to fresh TPM or TPM with 2% arginine, both at pH 7.4, was stable over the 4-day time period with no apparent degradation (control, not shown). Comparison of nonreduced BoNT levels from the very acidic pH group (pH 5.6) group with that of the mildly acidic pH group (pH 6.4) revealed a progressive decrease in BoNT levels until day 4, accompanied by an increase in smaller bands indicative of breakdown products (Fig. 7A and B). At about neutral pH (pH 7.2), an even stronger and faster decrease in BoNT levels was observed, concomitant with a greater increase in apparent breakdown products. Interestingly, at an alkaline pH of 8.0, the decrease in BoNT was less than at pH 7.2 and mirrored that at pH 6.4. While there are two predominant bands in the breakdown products that are similar in size to the 100-kDa HC and the 50-kDa LC of BoNTs, these bands were observed in nonreduced samples which would not allow for separation of HC and LC. In addition, detailed Western blot analyses with HC- and LC-specific antibodies indicated that the 100-kDa band is larger than the HC and contains the LC and a portion of the HC, whereas the apparent 50-kDa band is smaller than the LC and contains an HC fragment (data not shown). These data indicate proteolytic degradation of BoNT in a pH-dependent manner.

In order to investigate proteolytic involvement in toxin instability at pH 7.2, the same assay using spent 48-h culture supernatants of C. botulinum strain Hall A-*hyper* grown in TPM was performed at pH 7.2, and either a broad-spectrum protease inhibitor cocktail (PIC) or EDTA (metalloprotease inhibitor) was added before incubation. The PIC contained inhibitors of serine proteases, cysteine proteases, aspartic acid proteases, and aminopeptidases that are typically present in cell lysates. Quantitative Western blot analysis showed that the BoNT in nonreduced supernatant samples not treated with protease inhibitors decreased almost 2-fold by day 1 and 19-fold by day 4. Addition of PIC had no effect on this degradation, but addition of EDTA completely prevented protein degradation (Fig. 7C and D). These data indicate that a metalloprotease is responsible for pH-dependent protein degradation of BoNT/A in C. botulinum strain Hall A-*hyper* culture supernatant.

## DISCUSSION

The molecular mechanisms governing BoNT formation during growth of C. botulinum in various environments are not well understood, and the intricate steps involved have not been characterized. While several transcriptional control mechanisms have been identified as relatively minor regulators of BoNT production (14, 29, 30, 32–34), metabolic and environmental factors have been shown to strongly affect BoNT production (35–39). The data presented here for the first time suggest pH-dependent posttranslational proteolytic processing via a metalloprotease as a major mechanism of controlling BoNT production. The culture pH of C. botulinum cultures changes during growth in rich medium (34, 48) and is affected by environmental and nutritional factors that can alter the metabolism of the bacteria (39, 48). An acidic pH has been associated with greater toxin yield (48, 49), but the underlying mechanisms are unknown.

In a previous study, we had observed ∼1,000-fold suppression in active BoNT formation by C. botulinum if 2% arginine was added to complex medium during growth. In an attempt to understand the underlying mechanisms, the arginine catabolism pathway of C. botulinum strain Hall A-*hyper* was delineated using bioinformatics (Fig. 1). Second, select enzymes in the pathway were inactivated by ClosTron mutagenesis. Results from investigations into BoNT formation by the Hall A-*hyper* strain and these mutants in complex medium supplemented with either arginine or one of its metabolites indicated an indirect effect of arginine on BoNT formation (Fig. 2 and 3). Further, analysis of mRNA by Northern blotting indicated that the addition of 2% arginine into culture medium did not significantly alter transcription of the *bont* gene (Fig. 4).

During these studies, it became apparent that a drop in culture pH to acidic levels during growth correlated with greater toxin production, while an increase to alkaline pH levels correlated with decreased BoNT/A levels (see Table S1 and Fig. S1 in the supplemental material). Further analysis confirmed that C. botulinum strain Hall A-*hyper* cultures grown in TPM underwent a gradual decrease in pH during log phase, reaching a pH of ∼6 to 6.3, which stabilized at stationary phase and then increased very slightly with continued incubation (Fig. 5). Cultures grown in TPM supplemented with 2% arginine, on the other hand, underwent an increase in pH during log phase to alkaline levels of 7.5 to 7.8, which stabilized during stationary phase (Fig. 5). In addition, analysis of BoNT/A production during growth indicated an increase of intracellular BoNT/A until 24 h (late stationary phase) followed by a slight decrease of cell pellet-associated BoNT/A, which correlated with a gradual increase of BoNT/A in culture supernatants. This pattern is in agreement with toxin production during log phase and gradual release or secretion from the cells during the late log and lysis phases. In the cultures grown in TPM supplemented with arginine, on the other hand, almost no BoNT/A was detected in supernatant at any point, whereas cell pellet-associated BoNT/A increased throughout the 4-day study period. However, even cell pellet-associated BoNT/A levels were at least 5-fold reduced compared to those of cultures grown in TPM. Taken together, these data suggest posttranscriptional control of BoNT/A production in TPM with 2% arginine and indicate a potential correlation with culture pH.

To further examine the role of culture pH in BoNT/A production by C. botulinum strain Hall A-*hyper*, the culture pH during growth in TPM or TPM with 2% arginine was manually adjusted to alkaline levels instead of the normally acidic TPM culture, and to acidic levels for the normally alkaline TPM-2%-arginine cultures. This reversal in culture pH led to a reversal in BoNT/A production as well, with the now acidified TPM-2%-arginine culture producing normal levels of BoNT/A and the alkaline TPM culture resulting in barely detectable and largely degraded BoNT/A (Fig. 6). Similarly, pH reversal in in cultures of C. botulinum strain Hall A-*hyper argG*- or *arcB*- grown in TPM with 2% arginine confirmed correlation of decreased BoNT levels with alkaline pH. These data excluded a direct effect of arginine in controlling BoNT/A production and instead implicated culture pH as a crucial factor. Analyses of the effect of culture pH on the stability of BoNT/A in 48-h spent culture medium of C. botulinum strain Hall A-*hyper* grown in TPM further showed gradual degradation of BoNT/A at alkaline pH yet high stability at 37°C over 4 days at acidic pH (Fig. 7). Fresh TPM spiked with isolated BoNT/A complex resulted in no degradation at alkaline pH (data not shown), indicating the likely presence of a C. botulinum-produced protease in spent culture medium. In fact, addition of EDTA prevented BoNT/A degradation in spent culture medium (Fig. 7), further implicating a metalloprotease in toxin degradation.

These data suggest that BoNT/A production in proteolytic C. botulinum strain Hall A-*hyper* is strongly regulated on a posttranslational level by a pH-dependent metalloprotease that is produced during growth. The lower cell-associated BoNT/A levels at alkaline pH indicate that this proteolysis occurs inside and outside the bacterial cell. It has previously been described that Gram-positive bacteria adjust their intracellular pH based on extracellular pH changes (50). The metalloprotease involved in BoNT/A degradation is currently uncharacterized, and there are several possible mechanisms by which pH may play a role. First, most biologic enzymes are pH dependent and often show peak activity at neutral pH. In addition, pH-dependent conformational changes of the NTNH-BoNT/A1 complex causing instability at neutral or alkaline pH have been demonstrated (51), which could result in structural changes making the BoNT/A1 more susceptible to proteolytic attack by the metalloprotease.

Interestingly, the LC is a catalytically active Zn^2^-dependent metalloprotease, and the possibility that the metalloprotease(s) that is essential for production of biologically active BoNT in proteolytic C. botulinum is also involved in BoNT degradation cannot be ruled out (52, 53). BoNTs are produced as single 150-kDa polypeptides and are proteolytically converted to the more active dichain form of the 100-kDa HC and 50-kDa LC linked by a disulfide bond (8). The data show that BoNT/A in culture supernatants is mostly present in the “nicked” dichain form, while BoNT/A extracted from the cell pellet was only partially converted to the 100- and 50-kDa HC and LC, respectively (Fig. 5B). This indicates that both proteolytic processing and degradation of BoNT/A predominantly occur in the extracellular environment and raises the possibility that the same protease(s) may be responsible for both processes under different environmental conditions. In fact, a previous study has shown that BoNT/A and BoNT/E are degraded at 44°C by a protease with similar properties as the protease that activates BoNTs at lower temperatures, suggesting the possibility that it may be one protease with various functions at different temperatures (54). The data presented here are consistent with this study and suggest pH as an additional environmental or metabolically determined factor in controlling protease activity.

In conclusion, this study shows that the strong suppression of BoNT/A production in proteolytic C. botulinum by arginine is an indirect consequence of the catabolism of arginine by raising the pH to alkaline levels, most likely due to excess free ammonia production. Under normal (laboratory) growth conditions, proteolytic C. botulinum cultures acidify during growth to a pH of 5.7 to 6.2. The main effect of the alkaline pH appears to be the posttranslational proteolytic degradation of BoNT/A complex by a metalloprotease, although an additional effect on pretranslational regulation cannot be entirely excluded based on the results of this study. Both the protease, or proteases, involved in degradation and the molecular mechanisms underlying the pH dependence remain unknown, and future studies are required to resolve these outstanding questions. The findings from this study enhance our understanding of the mechanism(s) involved in BoNT formation by C. botulinum under various environmental or metabolic conditions, which is relevant for environmental and food safety, clinical efficacy, and production methods of pharmaceutical BoNTs.

## MATERIALS AND METHODS

### Ethics, biosafety, and biosecurity.

Our laboratory and personnel are registered with the Federal Select Agent Program for research involving botulinum neurotoxins (BoNTs) and BoNT-producing strains of clostridia. The research program, procedures, documentation, security, and facilities are closely monitored by the University of Wisconsin-Madison Biosecurity Task Force, the University of Wisconsin-Madison Office of Biological Safety, and the University of Wisconsin Select Agent Program, as well as the CDC and the Animal and Plant Health Inspection Service (APHIS) as part of the University of Wisconsin-Madison and CDC Select Agent Program. All personnel routinely receive suitability assessments and complete rigorous and continuing biosafety training, including biosafety level 3 (BSL3) and BSL2 select agent practices, before participating in laboratory studies involving BoNTs and neurotoxigenic C. botulinum. All animal studies conducted were approved by the University of Wisconsin-Madison IACUC.

### Strain, media, and growth.

The proteolytic C. botulinum strain Hall A-*hyper* (55), which produces BoNT/A1, was used in this study. Initial cultures for all growth experiments were started in anaerobic tryptone-peptone-glucose-yeast extract (TPGY) medium, pH 7.4 (5% Trypticase peptone [Difco], 2% yeast extract [Difco], 0.5% Bacto peptone [Difco], 0.4% glucose [Fisher Scientific], and 0.1% cysteine-HCl [MP Biomedicals]). Cultures were grown statically in nitrogen-flushed Hungate tubes or glass bottles at 35°C in an anaerobic chamber with an atmosphere of 80% N_2_, 10% CO_2_, and 10% H_2_ (Forma Anaerobic System, Marietta, OH), and all cell and toxin manipulations were conducted in the anaerobic chamber. Growth measurements (OD_600_) were determined in triplicate in an Eppendorf BioPhotometer.

For ClosTron mutagenesis experiments, reinforced clostridial medium (RCM) with 1% agar (both from Difco) and 2 μg/ml resazurin (as an indicator of anaerobic conditions) was used. Escherichia coli strain CA434 (56) was used as the donor strain for conjugation of the ClosTron plasmids into C. botulinum Hall A-*hyper*. E. coli cultures were grown aerobically at 37°C in a shaker at 225 rpm, in either Luria broth (LB) or SOC broth (57, 58). Antibiotics were used at the following concentrations: 20 μg/ml thiamphenicol, 12.5 μg/ml chloramphenicol, 25 μg/ml erythromycin, and 250 μg/ml cycloserine.

### Mouse bioassay.

An intraperitoneal (i.p.) time to death method was used to estimate toxicity of the cultures as previously described (39). In short, culture supernatant samples were treated with 5 μg/ml of trypsin (Worthington Biochemical Corporation) and incubated at 37°C for 1 h to proteolytically activate the BoNT. The treated culture supernatants were sterile filtered and mixed at a 1:1 ratio with GelPhos buffer (0.2% gelatin and 30 mM sodium phosphate, pH 6.3), and 0.5 ml per mouse was injected i.p. into groups of four female ICR mice (Harlan Laboratories). Survival time was recorded, and 50% lethal dose (LD_50_)/ml was estimated from the curve fit formula: *y* = 8334208.65 × 10^−0.025^*^x^* (39). Statistical analysis was performed with a paired Student *t* test (equal variance, two-tailed), and values were considered different with a *P* value of <0.05.

### ClosTron mutagenesis.

All ClosTron plasmids were designed using the Petruka algorithm on clostron.com (46, 59) and ordered from DNA2.0 (Atum.bio, Newark, CA). ClosTron mutagenesis was performed as previously described (60). Briefly, ClosTron plasmid DNA was transformed into E. coli strain CA434 and transferred by conjugation into C. botulinum strain Hall A-*hyper*, as previously described (46, 47, 60). The mating mixture of CA434 and Hall A-*hyper* was then plated onto RCM agar supplemented with cycloserine (to select for Hall A-*hyper*) and thiamphenicol (to select for acquisition of the ClosTron plasmid). Thiamphenicol-resistant colonies were restreaked on cycloserine- and erythromycin-supplemented media to select for Hall A-*hyper* mutants with the integrated intron and activated erythromycin resistance gene. Selected colonies were then serially restreaked onto erythromycin-supplemented medium plates to ensure purity and to cure the mutants of the ClosTron plasmid. Curing of the plasmid was verified by screening for loss of thiamphenicol resistance. Clones were analyzed by PCR using primers flanking the intron integration site (Table 1), such that mutants would produce a PCR product 1.8 kbp larger than wild type, due to integration of the intron. PCRs were performed using Phusion Master Mix with HF buffer (New England Biolabs) according to manufacturer’s instructions, and PCR products were separated on 1% Tris-acetate-EDTA (pH 8.0) gels and stained with ethidium bromide.

**TABLE 1:** Plasmids and primers used in this study

Plasmid or primer	Characteristic or sequence	Source
pMTL0007C-E2: ArcB-156a	ClosTron plasmid targeted to the Hall A-*hyper arcB* gene (CLC2465)	DNA2.0 (Atum)
pMTL0007C-E2: ArgG-147a	ClosTron plasmid targeted to the Hall A-*hyper argG* gene (CLC2544)	DNA2.0 (Atum)
ArcB-32F	CACTAATGGATTTCACACCAAAGGA	Integrated DNA Technologies
ArcB-507R	TGCCATATTATTAGCACCATCTCCA	
ArgG-18F	GCTTGCATACTCAGGAGGATTAGAT	Integrated DNA Technologies
ArgG-409R	TAGGATCTTGAGCCTTTACTCCAAC	
A1-500F	GCTTTGGACATGAAGTTTTGAATC	Integrated DNA Technologies
A1-926R (426 bp)	GAAGCAGTAGTACCTACTATTGATTTAGC	
A1-1026F	CAAAATGTTAACAGAGATTTACACAGAG	Integrated DNA Technologies
A1-1825R (799 bp)	GTTCTACCCAGCCTAAAAACATAG	
A1-1990F	CTGTTAGAATTTATACCAGAGATTGC	Integrated DNA Technologies
A1-2701R (711 bp)	CTATTGGATCAAAATTTACTTTACTACC	

### BoNT/A1 production studies.

To examine toxin production under various conditions, initially C. botulinum strain Hall A-*hyper* cultures were grown in TPGY overnight (∼18 h) to late log phase and used to inoculate experimental cultures at 1:100. For experimental determinations, cultures were grown at 35°C in TPM (2% casein hydrolysate [NZ Case TT; Kerry Ingredients], 1% yeast extract [Difco], and 0.5% glucose [Fisher Scientific]), pH 7.4, or in TPM supplemented with 0.115 M arginine (TPM+Arg), 0.115 M citrulline (TPM+Cit), or 0.115 M ornithine (TPM+Orn), respectively (all from Sigma-Aldrich).

For studies examining culture pH and toxin formation, cultures were grown as described above in the indicated medium, and pH was measured at the same time as toxin production. For cultures in which the pH was examined throughout growth, cultures were grown in 200-ml Pyrex glass bottles with 150 ml TPM and TPM with 0.115 M (2%) arginine in the anaerobic chamber, and 1-ml aliquots were removed periodically throughout the 96-h incubation for absorbance and pH measurements. In assays where manual pH adjustments were made, pH of cultures was adjusted inside the anaerobic chamber by adding either 4 M NaOH or 5 M HCl between 10 and 18 h postinoculation to maintain the desired pH ranges. The times of acid or base addition and resulting pH are indicated in Results.

### Toxin stability in spent culture medium.

Triplicate samples of strain Hall A-*hyper* were grown in 10 ml or in 150 ml TPM at 37°C for 48 h as indicated in Results. At 48 h postinoculation, 10-ml or 25-ml aliquots of each culture were centrifuged for 10 min at 6,000 × *g* and the toxin-containing supernatants were collected. Supernatants were split into 4 equal samples, and the pH of the samples was adjusted to either 8.0, 7.2, 6.4, or 5.6 by adding either 1 M NaOH or 5 M HCl for each of the triplicate cultures. The pH-adjusted supernatant aliquots were sterile filtered using 0.22-μm polyvinylidene difluoride (PVDF) filters and either used directly or with the following additions as indicated: protease inhibitor cocktail (EDTA-free) (PIC) {Pierce catalog no. A32955, containing AEBSF [4-(2-aminoethyl)benzenesulfonyl fluoride hydrochloride], aprotinin, bestatin, E-64, leupeptin, and pepstatin A}, PIC plus 5 mM EDTA, 5 mM EDTA, or no treatment. All samples were incubated at 37°C for up to 4 days, and samples were taken at 0, 24, and 96 h for comparative analysis by SDS-PAGE and Western blotting. Controls included fresh TPM and TPM with 2% arginine, both at pH 7.4.

### SDS-PAGE analyses and Western blots.

For SDS-PAGE analysis, either whole culture, culture supernatant, or cell lysates were analyzed as indicated in Results. For whole-culture samples, culture suspensions containing both cells and supernatant were mixed, 1/4 volume of 4× Laemmli sodium dodecyl sulfate (SDS) sample buffer (8% SDS, 40% glycerol, 0.008% bromophenol blue, 0.05 M EDTA, and 0.25 M Tris HCl, pH 6.8) was added, and the samples were immediately heated to 70°C for 10 min. Culture supernatants and cell pellets were obtained by centrifugation for 10 min at 16,000 × *g*. Cell pellets were resuspended in an equal volume of TES buffer (25 mM Tris-HCl, pH 8.0, 0.1 M NaCl, and 0.001 M EDTA) containing lysozyme (0.02 g/ml), and cells were lysed for 10 min at 35°C. SDS sample buffer was added to both the treated pellet and the supernatant fractions, and samples were heated to 70°C for 10 min. Samples were separated on Life Technologies 4 to 12% Bis-Tris gels, in NuPAGE morpholineethanesulfonic acid (MES) SDS running buffer (Life Technologies), and transferred to a 0.45-μm PVDF membrane (Immobilon-P). Immunoblot assays used the Western Breeze solutions (Life Technologies) and anti-BoNT/A1 polyclonal rabbit primary antibodies prepared in our laboratory (61) and anti-rabbit IgG-alkaline phosphatase-conjugated secondary antibody produced in goat (Sigma). Bands were visualized using chemiluminescent PhosphaGLO AP substrate (KPL) and obtained using a Fotodyne Foto/Analyst FX imager. Densitometry comparing samples on the same membrane was conducted using TotalLab Quant. Average and standard deviations for at least triplicate samples were determined in Excel. A Student *t* test (paired, equal variance, *n* = 3) was conducted on the triplicate samples, and data points were considered significantly different at a *P* value of <0.05.

### Northern hybridization.

C. botulinum strain Hall A-*hyper* cultures were grown in TPM or TPM plus 0.115 M (2%) arginine. Culture aliquots were removed from the growth flasks inside the anaerobic chamber and mixed with 2 volumes of RNA-Protect bacterial reagent (Qiagen) for 5 min at 25°C. Treated cultures were removed from the anaerobic chamber, and bacterial cells were collected by centrifugation for 10 min at 5,000 × *g*. Culture supernatant was decanted, and tubes with bacterial pellet were flash-frozen in liquid nitrogen and stored at −80°C until RNA preparation. Bacterial pellets were resuspended in TESS buffer (20 mM Tris-HCl, pH 8.0, 10 mM EDTA, 6.7% sucrose, and 0.05% SDS) containing lysozyme (20 mg/ml) and proteinase K (10 mg/ml) and incubated for 15 min at 25°C. Cell lysates were mixed with TRIzol-LS reagent (Invitrogen) preheated to 65°C and incubated at 25°C for 10 min, followed by centrifugation at 12,000 × *g* for 10 min at 4°C. Cell lysates were transferred to PhaseMaker tubes (Invitrogen) containing BCP reagent (1-bromo-3-chloropropane), and contents were vortexed for 30 s, incubated at 25°C for 10 min, and centrifuged at 12,000 × *g* for 10 min at 4°C. RNA was purified from the aqueous phase using Qiagen RNeasy mini spin columns following manufacturer’s instructions.

Isolated RNA (5 μg/lane) was electrophoresed through 1.2% agarose gels containing 0.6 M formaldehyde in 1× MOPS buffer (3-[*N*-morpholino] propanesulfonic acid) and then stained with ethidium bromide (1 μg/ml). RNA was transferred to an H^+^ nylon membrane (Amersham Hybond XL) from 10× SSC (1× SSC is 0.15 M NaCl plus 0.015 M sodium citrate) using a downward capillary transfer system (Turboblotter; Whatman). RNA was fixed to the membrane by heating at 80°C for 30 min on a gel dryer (Bio-Rad Laboratories, Hercules, CA). Membranes were prehybridized for 3 h at 68°C and hybridized for 16 h at 68°C in ULTRAhyb (ultrasensitive hybridization buffer; Invitrogen) using ^32^P-labeled antisense RNA probes of the BoNT/A1 gene prepared as described below. Excess probe was removed by two 5-min washes with 2× SSPE (1× SSPE = 150 mM NaCl, 10 mM NaH_2_PO_4_, 1 mM EDTA, pH 7.4) and 0.1% SDS at 25°C and two 60-min washes with 0.1× SSPE, 0.1% SDS at 68°C. Membranes were exposed for 1 to 2 h to a PhosphorImager screen, and the capture and quantification of the hybridization signals were performed using a Typhoon FLA 9000 imaging scanner and ImageQuant software (GE Healthcare Bio-Sciences Corp., Piscataway, NJ). Relative *ntnh*-*bont* bicistronic and monocistronic *bont* mRNA levels were measured together. The mRNA expression levels were normalized to the levels of the TPM cultures grown for 12 h, which were defined as 100%. Average and standard deviations from triplicate samples were determined.

### Hybridization probes.

PCR primers (IDT Technologies) were used to amplify three different regions of the BoNT/A1 gene (Table 1). C. botulinum strain Hall A-*hyper* chromosomal DNA was used as a template. PCRs were performed using Phusion high-fidelity PCR master mix with high-fidelity buffer (New England Biolabs Inc., Ipswich, MA) in a ProFlex PCR cycler (Applied Biosystems by Life Technologies, Bedford, MA). The PCR fragments were purified from a 1% agarose gel using the Monarch DNA gel extraction kit (New England Biolabs Inc., Ipswich, MA) following manufacturer’s instructions. Fragments were inserted into pJET1.2 vector using the CloneJET PCR cloning kit (ThermoFisher Scientific, Waltham, MA), and DNA sequences were confirmed by DNA sequencing (University of Wisconsin-Madison Biotechnology Center). Clones of BoNT/A1 gene fragments inserted downstream of the T7 promoter in the reverse orientation of the BoNT gene were used for generation of antisense RNA probes. The plasmids were linearized with restriction enzyme XbaI, the enzyme was inactivated for 20 min at 65°C, and linearized plasmids were then treated with DNA polymerase I (Klenow fragment) (New England Biolabs Inc., Ipswich, MA) in the presence of deoxynucleoside triphosphates (dNTPs) to generate blunt ends of the DNA molecules. The linearized DNA templates were purified (Monarch PCR and DNA cleanup kit; New England Biolabs Inc., Ipswich, MA) following manufacturer’s instructions. ^32^P-labeled antisense RNA hybridization probes were generated from the three BoNT/A1 gene fragments using the HiScribe T7 high-yield RNA synthesis kit (New England Biolabs Inc., Ipswich, MA) and [^32^P]UTP (3,000 Ci/mmol: PerkinElmer, Inc.) following manufacturer’s instructions. Unincorporated nucleotides were removed from the synthesized probes by spin column chromatography (NucAway columns; Invitrogen) according to manufacturer’s instructions. All three probes were combined and used at 1.5 × 10^6^ cpm/ml in hybridization with RNA membranes. Oligonucleotide primers used for amplification of BoNT gene fragments are shown in Table 1.

### Statistics.

All values were measured in at least triplicate values, and values are expressed as average ± standard deviation (SD). Differences between samples were assessed using paired Student’s *t* test where statistical significance is assumed for *P* < 0.05.
