# Supplementary figures and images for: Posttranslational Regulation of Botulinum Neurotoxin Production in Clostridium botulinum Hall A-hyper
Source: mSphere. 2021 Aug 4;6(4):e00328-21. doi: 10.1128/mSphere.00328-21 (PMC8386421; doi:10.1128/mSphere.00328-21)

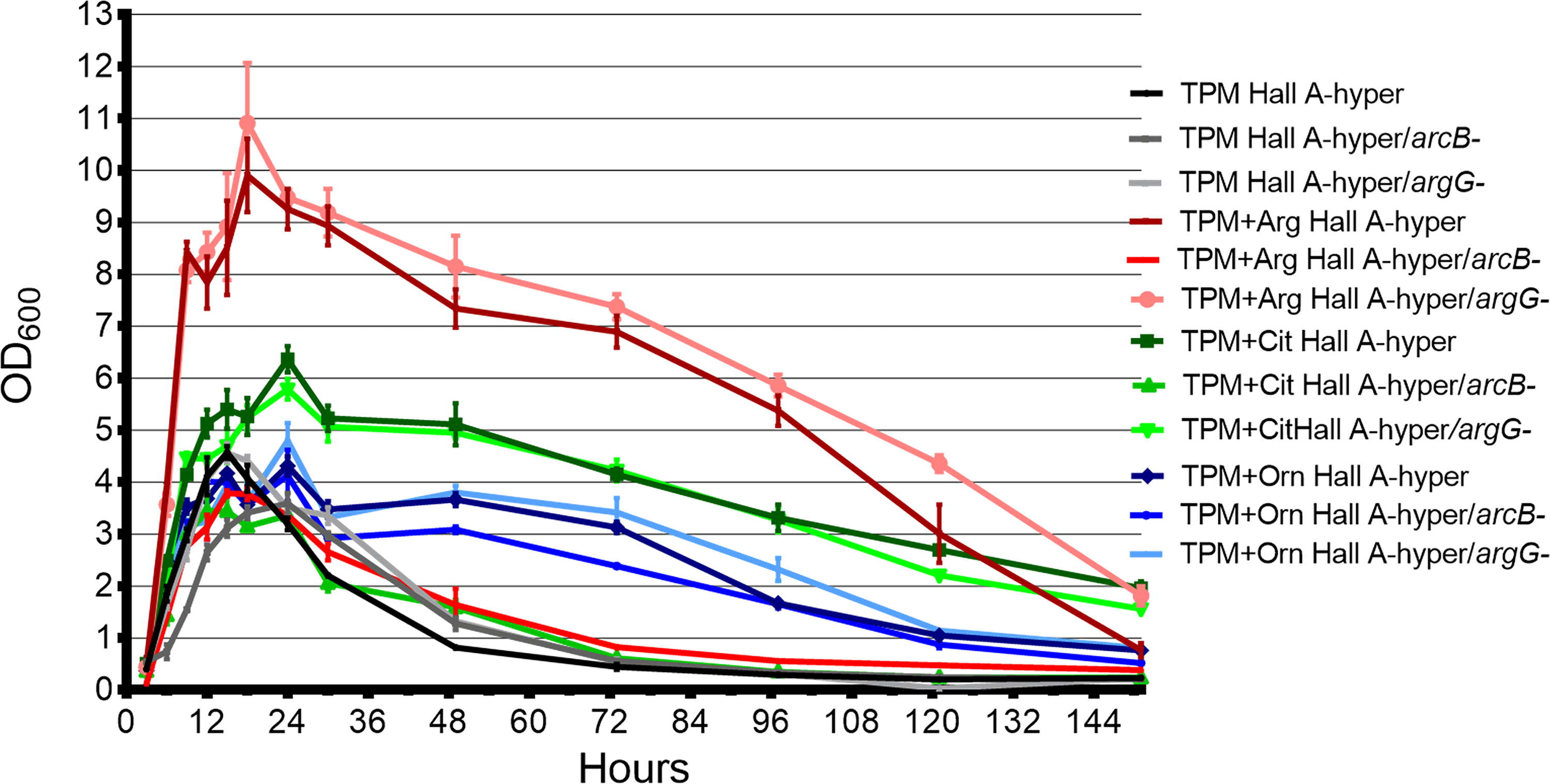

Supplement: FIG S1 [file msphere.00328-21-sf001.tif]

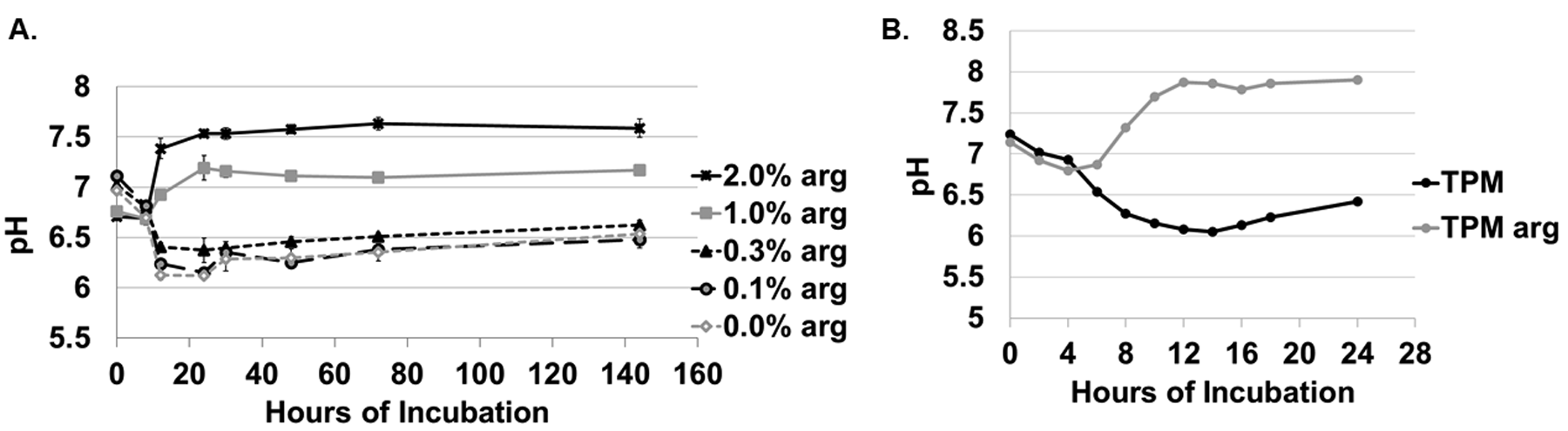

Supplement: FIG S2 [file msphere.00328-21-sf002.tif]
