# Supplementary material for: Posttranslational Regulation of Botulinum Neurotoxin Production in Clostridium botulinum Hall A-hyper
Source: mSphere. 2021 Aug 4;6(4):e00328-21. doi: 10.1128/mSphere.00328-21 (PMC8386421; doi:10.1128/mSphere.00328-21)
Supplement: TABLE S1 [file msphere.00328-21-st001.docx]

|  | **Strain** | **24 h pH** | **144 h pH** |
| --- | --- | --- | --- |
| **TPM** | Hall A-hyper | 5.9 | 6.5 |
|  | Hall A-hyper/argG^-^ | 5.9 | 6.5 |
|  | Hall A-hyper/arcB^-^ | 5.7 | 6.3 |
| **TPM+Arg** | Hall A-hyper | 7.4 | 7.8 |
|  | Hall A-hyper/argG^-^ | 7.5 | 7.8 |
|  | Hall A-hyper/arcB^-^ | 5.4 | 5.9 |
| **TPM+Cit** | Hall A-hyper | 6.9 | 7.7 |
|  | Hall A-hyper/argG^-^ | 6.8 | 7.7 |
|  | Hall A-hyper/arcB^-^ | 5.9 | 6.4 |
| **TPM+Orn** | Hall A-hyper | 5.8 | 6.3 |
|  | Hall A-hyper/argG^-^ | 5.8 | 6.3 |
|  | Hall A-hyper/arcB^-^ | 5.6 | 6.1 |
